# Respiratory dysbiosis in cats with spontaneous allergic asthma

**DOI:** 10.3389/fvets.2022.930385

**Published:** 2022-09-08

**Authors:** Aida I. Vientós-Plotts, Aaron C. Ericsson, Zachary L. McAdams, Hansjorg Rindt, Carol R. Reinero

**Affiliations:** ^1^College of Veterinary Medicine, University of Missouri, Columbia, MO, United States; ^2^Department of Veterinary Medicine and Surgery, College of Veterinary Medicine, University of Missouri, Columbia, MO, United States; ^3^Comparative Internal Medicine Laboratory, College of Veterinary Medicine, University of Missouri, Columbia, MO, United States; ^4^University of Missouri Metagenomics Center, University of Missouri, Columbia, MO, United States; ^5^Department of Veterinary Pathobiology, College of Veterinary Medicine, University of Missouri, Columbia, MO, United States

**Keywords:** respiratory microbiota, inflammatory airway disease, large animal model, 16S rRNA gene, translational research

## Abstract

Deviations from a core airway microbiota have been associated with the development and progression of asthma as well as disease severity. Pet cats represent a large animal model for allergic asthma, as they spontaneously develop a disease similar to atopic childhood asthma. This study aimed to describe the lower airway microbiota of asthmatic pet cats and compare it to healthy cats to document respiratory dysbiosis occurring with airway inflammation. We hypothesized that asthmatic cats would have lower airway dysbiosis characterized by a decrease in richness, diversity, and alterations in microbial community composition including identification of possible pathobionts. In the current study, a significant difference in airway microbiota composition was documented between spontaneously asthmatic pet cats and healthy research cats mirroring the finding of dysbiosis in asthmatic humans. *Filobacterium* and *Acinetobacter* spp. were identified as predominant taxa in asthmatic cats without documented infection based on standard culture and could represent pathobionts in the lower airways of cats. *Mycoplasma felis*, a known lower airway pathogen of cats, was identified in 35% of asthmatic but not healthy cats.

This article has been published alongside “Temporal changes of the respiratory microbiota as cats transition from health to experimental acute and chronic allergic asthma” (1).

## Introduction

In humans, asthma is used to describe a group of inflammatory airway diseases with distinct phenotypes based on the clinical manifestation of the disease and underpinned by specific endotypes or mechanistic pathways (2). Interactions between the host immune system and microbiome have recently been recognized as important contributors to asthmatic endotypes, driving studies of the composition and structure of the airway microbiota of asthmatic patients to develop a deeper understanding of such a complex disease (3–6). While the relationship among phenotypes, endotypes, and the respiratory microbiota is not completely understood (7–9), the respiratory microbiota is known to play critical roles in the development, regulation, and maintenance of a healthy immune system (10–12).

Further, dysbiosis or alterations in the composition of the microbial community can contribute to immune dysregulation, potentially influencing the development of disease as well as response to treatment (7, 13–15). In a healthy environment, equilibrium or homeostasis exists, and triggers such as environmental irritants may lead to pathogen colonization or alterations in microbial community composition that cause low-grade inflammation, allowing for bacterial replication, epithelial damage, and recruitment of immune cells, contributing to a vicious cycle of inflammation. It is unclear if the dysbiosis documented in asthma occurs as a result of inflammation or secondary bacterial infection, or if dysbiosis occurs first, triggering inflammation, and providing a niche for opportunistic pathogens or pathobionts to thrive, or both (16).

Differences in microbial community composition between spontaneously asthmatic and healthy airways of humans (5, 17) and horses (18, 19) as well as experimentally induced asthma in mice (20) and cats (1) have been documented. Since cats can experimentally or spontaneously develop airway eosinophilia, airway hyperresponsiveness, and airway remodeling analogous to human allergic asthma, they serve as an important large animal model for the type 2 high endotype (21, 22). In this study, we aimed to characterize the lower airway microbiota of pet cats with spontaneous allergic asthma and compare it to that of healthy cats. We hypothesized that, as in humans, spontaneously asthmatic pet cats would have decreased richness and diversity and significant alterations in the microbial community, and the potential emergence of possible pathobionts compared to healthy cats.

## Materials and methods

### Ethics statement

All studies were performed in accordance with the Guide for the Use and Care of Laboratory Animals and were approved by the University of Missouri Institutional Animal Care and Use Committee (MU IACUC protocol #7891).

### Cats

Client-owned pet cats presenting to the University of Missouri Veterinary Health Center between 2015 and 2019 that were diagnosed with asthma and had bronchoalveolar lavage fluid (BALF) samples banked were retrospectively enrolled. Diagnostic criteria for feline asthma included a history of clinical signs such as cough and respiratory distress, thoracic radiographs with evidence of a bronchial pattern and lung hyperinflation, and BALF cytology containing >7% eosinophils (23). Signalment, home environment (indoor vs. outdoor), clinical signs, peripheral eosinophil count, heartworm test results, serum allergy testing, thoracic radiographic pattern, and BALF cytology report were extracted from the medical record when available. A clinical severity score was assigned based upon a previously published (24) three-point scoring system, namely, 1 = mild (cough alone); 2 = moderate (wheezing or exercise intolerance); or 3 = severe (respiratory distress episodes).

Banked BALF samples from eleven healthy research cats were used as controls. Control cats were from a research colony (Comparative Internal Medicine Laboratory, University of Missouri, Columbia, MO). Cats were housed in large runs with elevated platforms for climbing and enrichment toys. Access to food and clean drinking water was provided *ad libitum*. There were 6 female and 5 male cats from 2 different litters and they were housed according to sex. Cats were determined to be healthy by the absence of respiratory clinical signs, a normal physical examination by a board-certified veterinary internal medicine specialist, and a lack of cytologic evidence of infection or inflammation from BALF samples. Euthanasia was not an endpoint of the study; all cats were subsequently adopted into private homes.

### Sample collection

All cats, control and client-owned, were anesthetized and carefully intubated using a sterile 3.5–4 French endotracheal tube. To collect BALF, a 20 ml aliquot of sterile saline was instilled and aspirated *via* a sterile 8 French red rubber catheter that was threaded through the endotracheal tube until it was gently wedged in the distal airway. Immediately after collection, all samples were placed on ice and transported to the laboratory. Promptly after collection, samples were centrifuged to pellet bacterial cells. The supernatant was discarded and pellets were resuspended in 800 μl of lysis buffer adapted from Yu et al. (4% sodium dodecyl sulfate, 50 mM EDTA, 500 mM NaCl, and 50 mM Tris–HCl pH 8.0) (25). All the samples were banked at −80 °C until the end of the study, and DNA was extracted as a single batch.

### DNA extraction, 16S rRNA library preparation, sequencing, and informatics

DNA from BALF was extracted using the column method as previously described (26, 27). Library construction and sequencing were completed at the University of Missouri DNA Core facility as previously described (26). Assembly, filtering, binning, and annotation of DNA sequences were performed at MU Informatics with Quantitative Insights Into Microbial Ecology 2 (QIIME 2) v2021.2 (28). Sequences were trimmed from the Illumina adapters with cutadapt (29). Using DADA2 (30), trimmed forward and reverse reads were truncated to 150 base pairs, paired, and then denoised into unique sequences called Amplicon Sequence Variants (ASVs). A feature table containing the frequency of each ASV per sample was rarefied to 11,562 total features per sample, maximizing the number of subsampled features per sample and a total number of samples retained for further analysis. Samples with a total feature number of <1,488 were omitted from downstream analyses. A taxonomy was assigned to each unique ASV with a sklearn algorithm (31) using the QIIME2-provided 99% non-redundant SILVA v132 reference database of the 515F/806R region of the 16S rRNA gene (28, 32). Code used for sequence processing can be accessed at https://github.com/mubioinformatics/nf-qimme2 and https://github.com/ericsson-lab/spontaneous_asthma. Alpha diversity analysis, principal coordinate analysis (PCoA), and one-way permutational analysis of variance (PERMANOVA) of Jaccard distances (1/4 root-transformed data) were performed using the vegan v2.5-7 (33) package within R v4.1.2 (34).

### Statistical analysis

Statistical analysis was performed using Sigma Plot 14.0 (Systat Software Inc., Carlsbad, CA) and R v4.1.2. Normality was first tested using the Shapiro–Wilk method, and equal variance was tested using the Brown–Forsyth method. Fischer's exact tests were utilized to determine associations. Mann–Whitney Rank sum tests were used to test for differences between sample sites in coverage, richness, and relative abundance of all taxa at the level of family detected at > 0.5% of samples. Results are presented as mean ± SEM. PCoA was used to visualize the relatedness of samples. Enhanced Volcano (35) was utilized to generate a volcano plot to visualize which taxa had undergone the most significant changes. Results were considered statistically significant for *p*-values ≤ 0.05.

## Results

### Cats

Twenty-six cats, 12 females and 14 males, all altered, weighing 4.9 ± 0.19 kg (mean ± SEM) from a variety of breeds including domestic short hair (*n* = 18), domestic long hair (3), Siamese (2), Persian, (1), Maine Coon (1), and Himalayan (1) were enrolled. Clinical signs on presentation were reported to be acute (<2 weeks; 6) or chronic (>2 weeks; 20) and included cough (15), wheeze (6), respiratory distress (5), increased respiratory effort in combination with the increased respiratory rate (5), increased respiratory effort alone (3), and exercise intolerance (3). Cats were categorized as having mild (12), moderate (7), or severe (7) signs based on the clinical severity score. Ten cats also had upper airway signs, including nasal discharge (5), sneezing (3), and stridor (2). In addition to asthma, the cats with upper airway signs were also diagnosed with chronic rhinitis (3), nasopharyngeal stenosis (2), and nasal carcinoma (1). None of the cats tested for heartworm antibodies were positive (*n* = 16). BALF culture results were negative in 21/24 and were not performed in 2/26 cats. In 3 cats, culture was positive for *Stenotrophomonas maltophilia (Pseudomonas maltophilia), Pseudomonas putida*, and an unidentified *Pasteurella* spp. and *Streptococcus* spp. *Pasteurella multocida*, and *Pseudomonas aeruginosa*. Recent drug administration, defined as within 14 days prior to sampling, included antibiotics (4), corticosteroids (4), or both (2). The 11 control cats were significantly younger than the asthmatic cats (2.7 ± 0.1 vs. 5.2 ± 0.6 years, respectively; *p* = 0.016). BALF eosinophil counts in healthy cats ranged from 2.5% to 6.5% (mean ± SEM; 4.6 ± 0.5%) and were significantly lower compared to asthmatic cats, which ranged from 11.1% to 71.5% (37.8 ± 3.8; *p* < 0.001). Individual cat data are summarized in Supplementary Table 1.

### Coverage and richness in BALF are significantly lower and diversity higher in asthmatic vs. healthy cats

**Figure 1:**
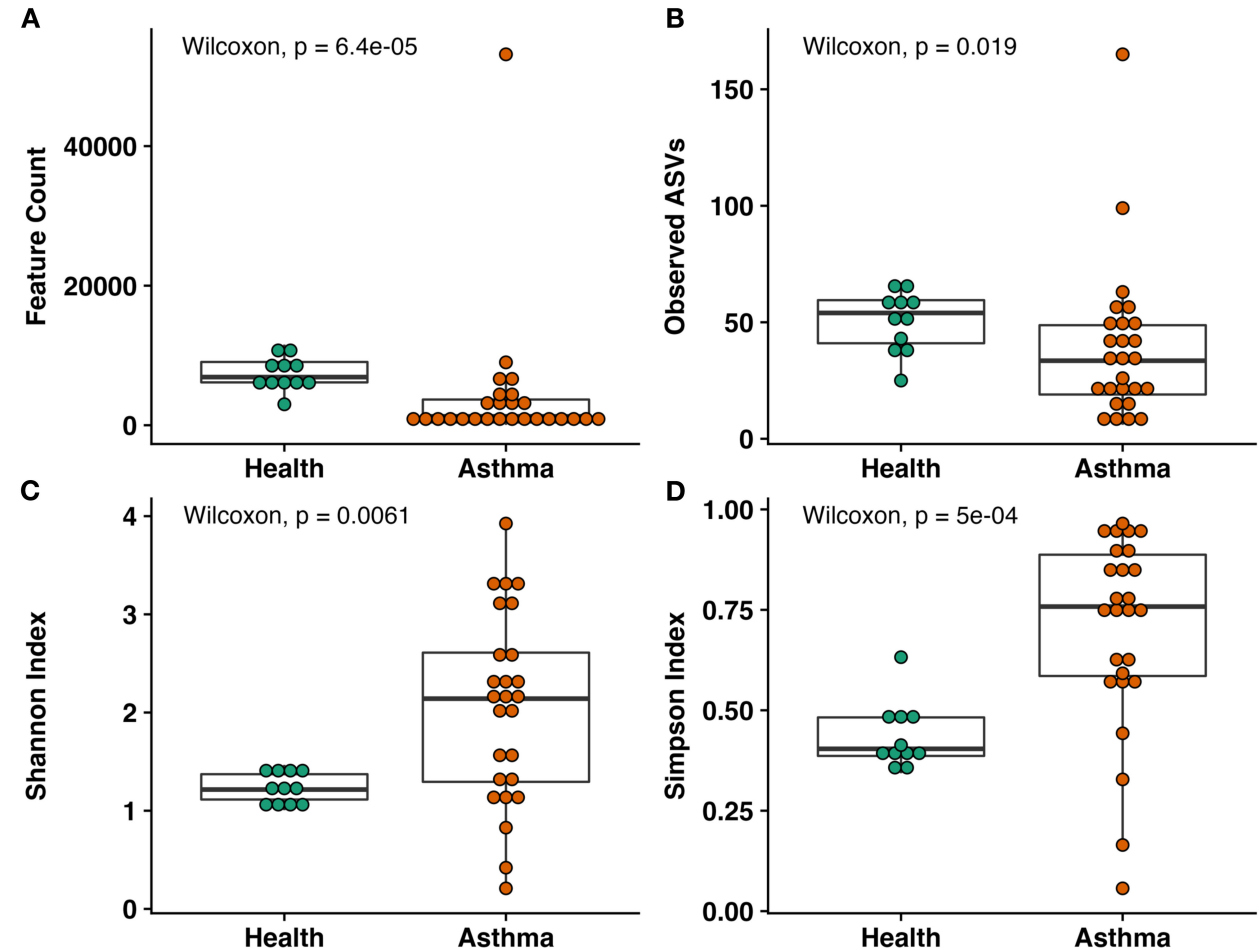
Alpha diversity metrics including **(A)** total observed features, **(B)** within sample richness, **(C)** Shannon Index, and **(D)** Simpson Index. Significant decreases in coverage and richness were observed in asthma compared to healthy cats. Within sample diversity was significantly increased in asthmatic cats compared to healthy cats. Wilcoxon rank sum test, *p*-values indicated.

Samples of BALF from asthmatic cats had significantly lower depth of coverage than healthy cats (mean ± SEM of 2,172 ± 92 and 7,441 ± 216 sequences/sample, respectively, *p* < 0.001), as well as richness (33 ± 1 and 50 ± 1, respectively; *p* = 0.019; Figures 1A,B). Asthmatic cats exhibited higher diversity than healthy cats using Shannon (2.05 ± 9.19 and 1.24 ± 0.05, respectively; *p* = 0.0061) and Simpson (0.70 ± 0.05 and 0.44 ± 0.02, respectively; *p* < 0.001; Figures 1C,D).

### Microbial community composition is significantly altered in asthmatic cats

**Figure 2:**
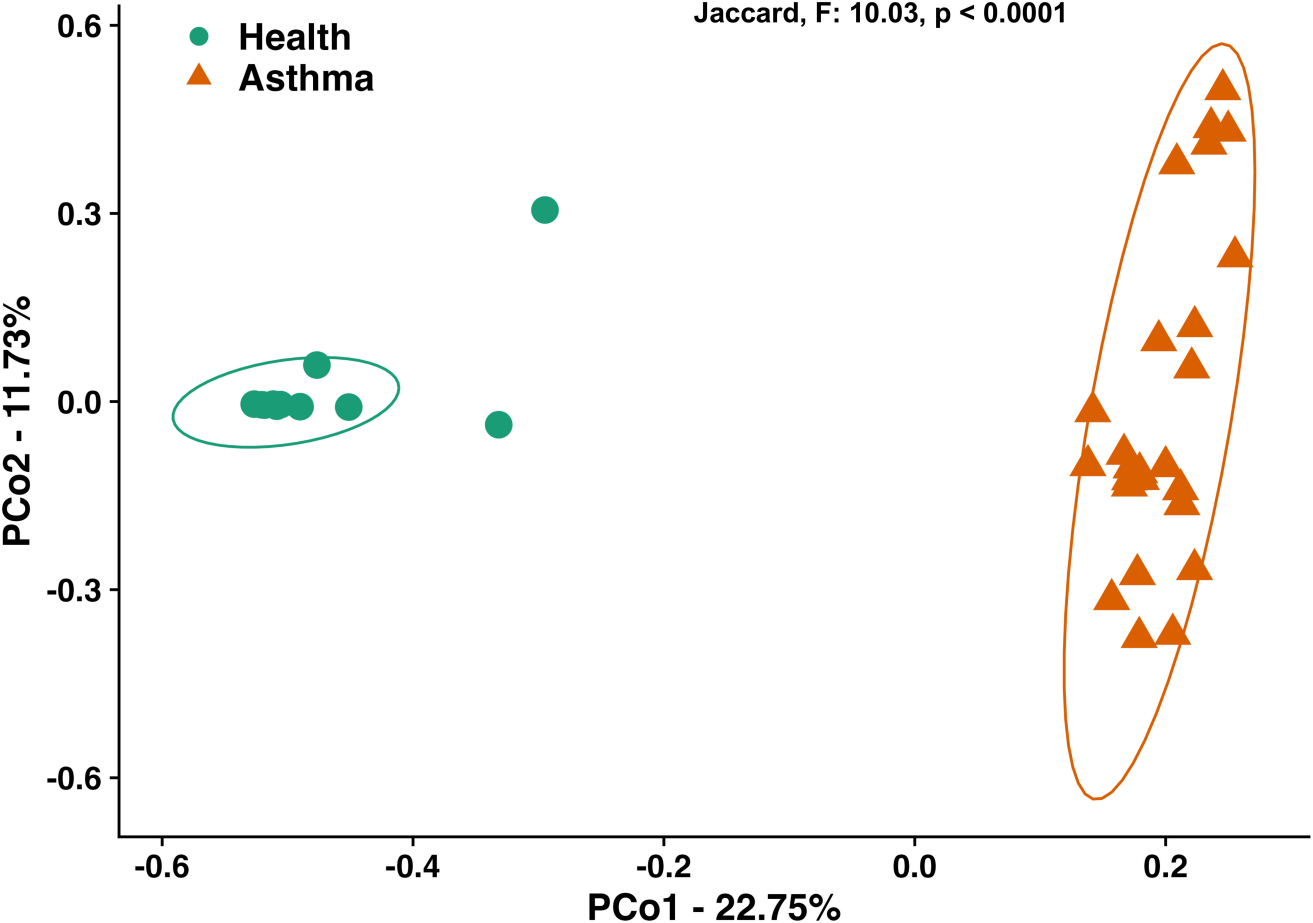
Principal coordinate analysis of Jaccard similarity index showing significant differences (*p* < 0.0001; *F* = 10.03) in microbial community composition between healthy and spontaneously asthmatic cats. Circles represent healthy cats; triangles represent asthmatic cats and ellipses represent 95% CIs.

**Figure 3:**
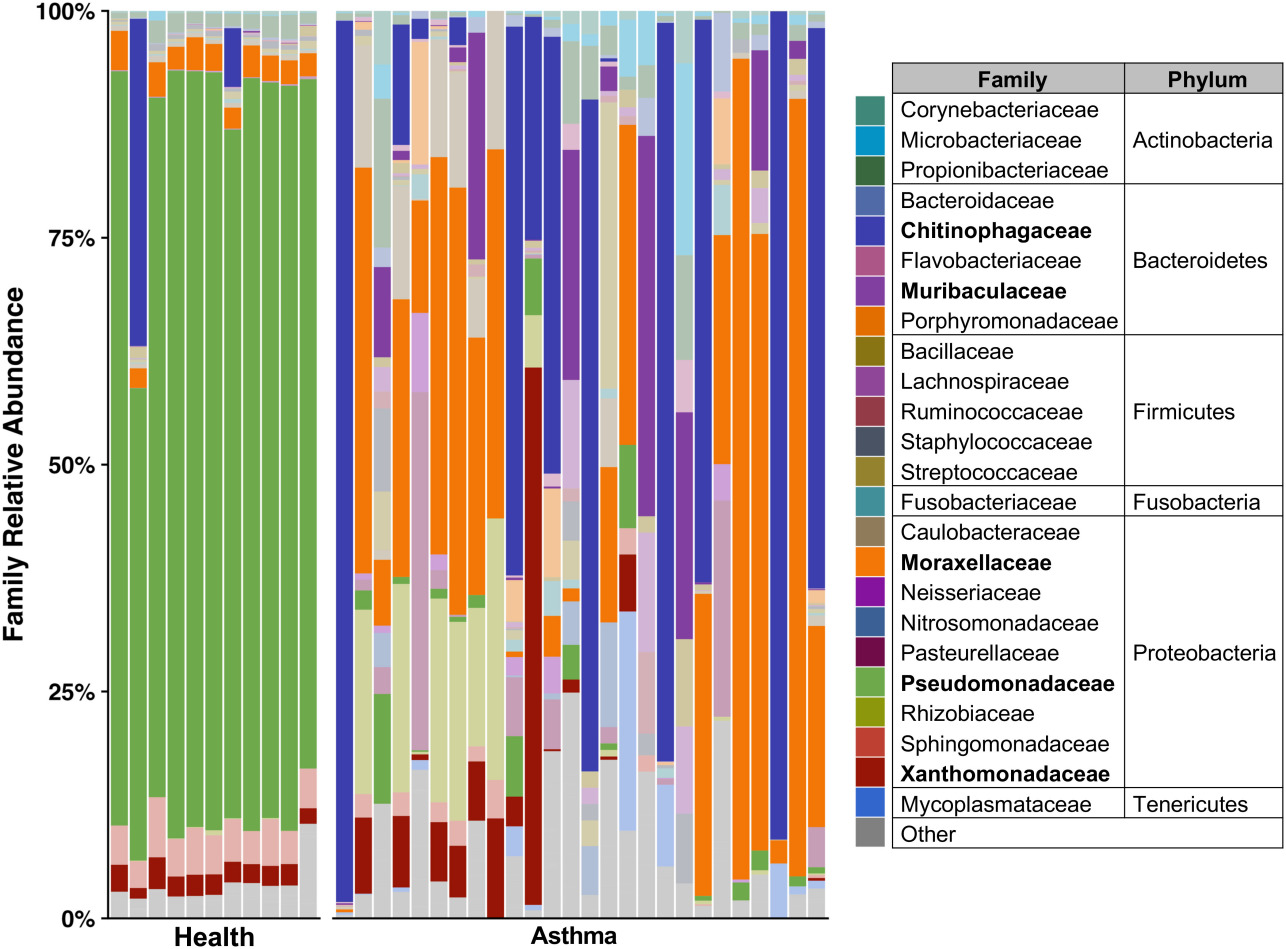
Taxa present in bronchoalveolar lavage fluid—mean relative abundance of taxa present at > 5% in bronchoalveolar lavage fluid collected from healthy and cats with spontaneous asthma. Families with a relative abundance of <5% are grouped into “Other.” Families with >40% relative abundance in at least one sample are bolded in the legend and represented by a darker color in the plot.

**Figure 4:**
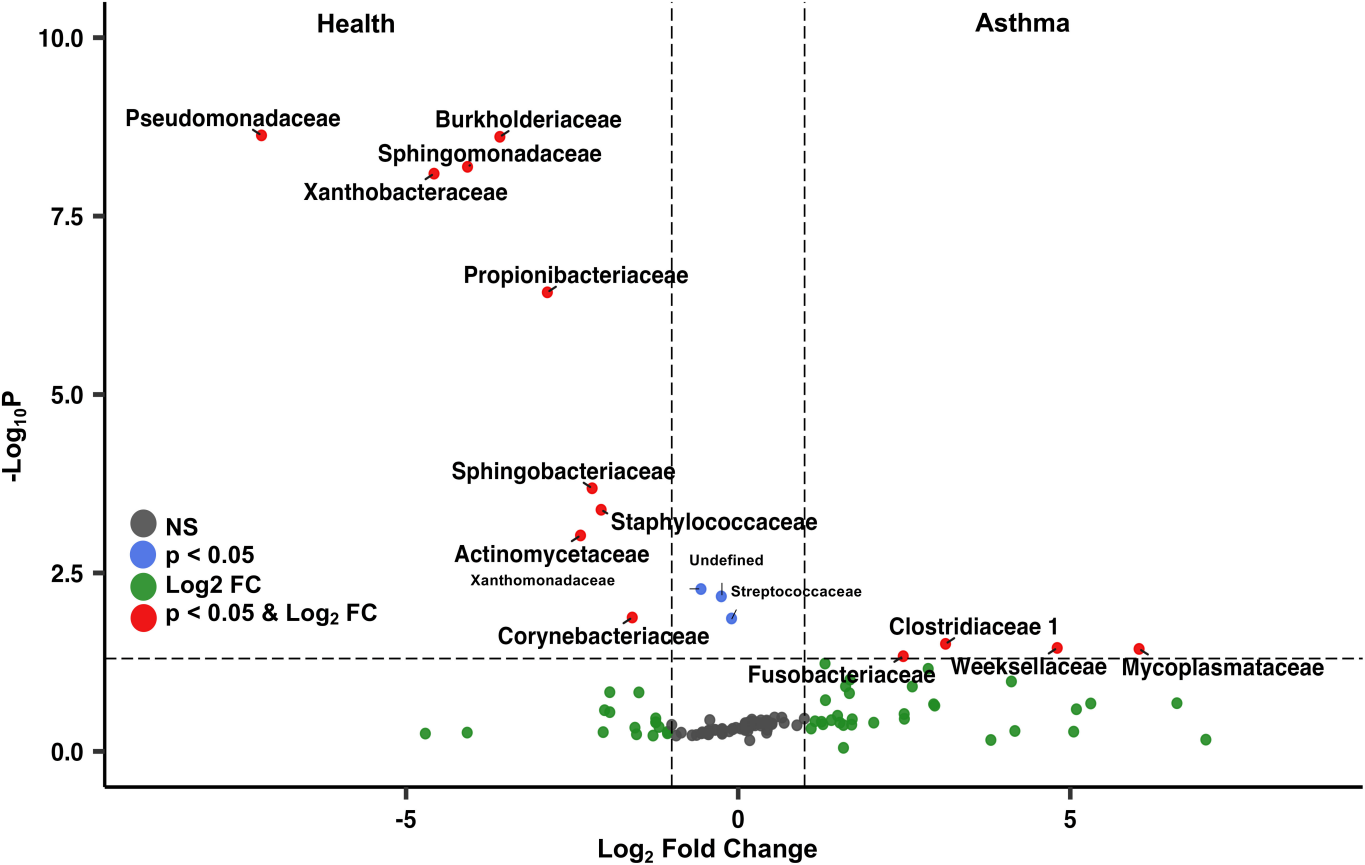
Volcano plot highlighting the most abundant families in the lower airways that were significantly different (*p* < 0.05) and underwent at least a 2-fold change in abundance in asthmatic pet cats compared to healthy cats. The taxa on the top left were more abundant in health, whereas the taxa on the right were more abundant in asthmatic pet cats.

**Figure 5:**
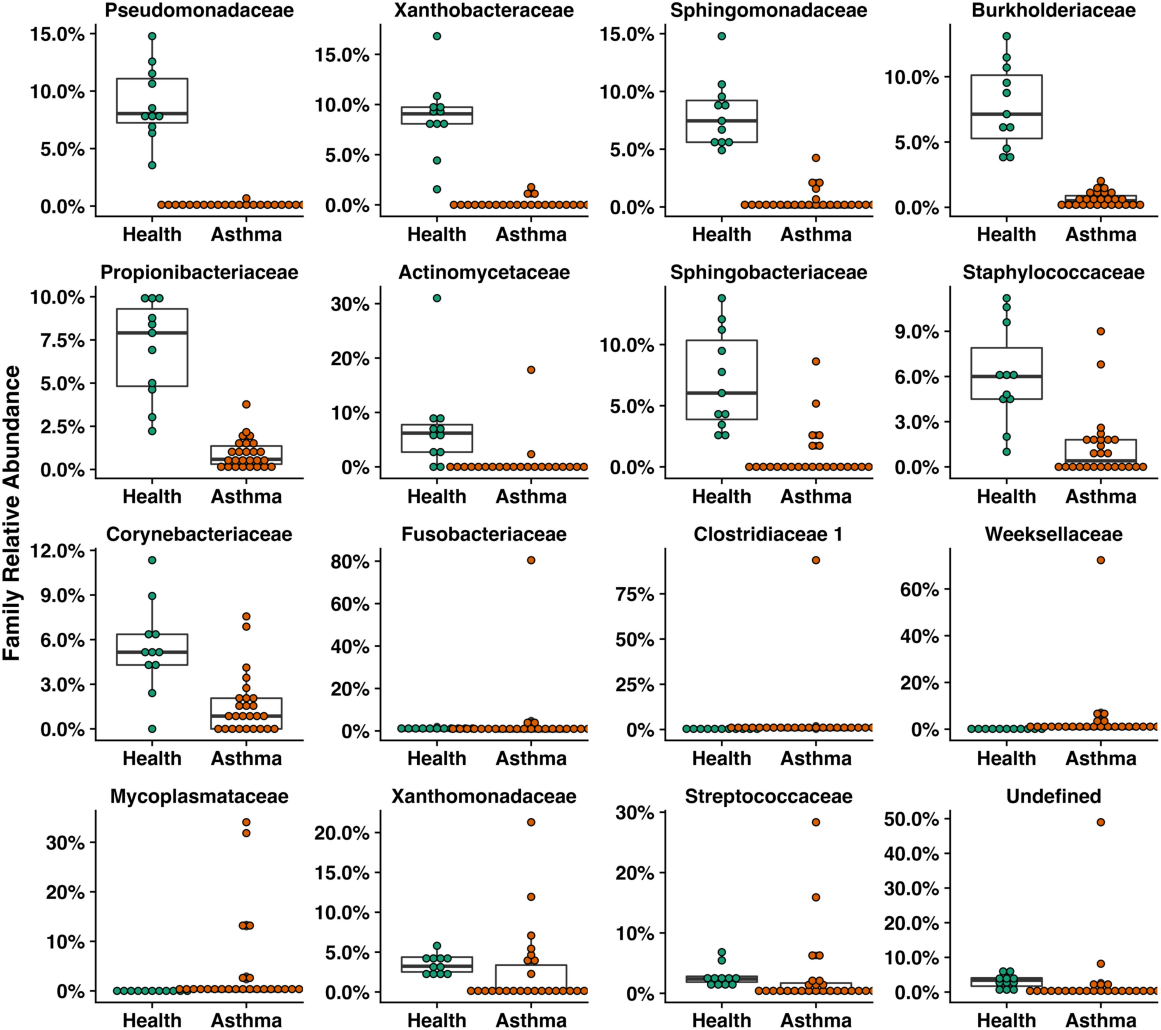
Box plots of families that were significantly different including those with a greater than two-fold change or were significantly different in healthy compared to asthmatic pet cats.

Principal coordinate analysis (PCoA), used to assess the β-diversity of microbial communities, showed no overlap between healthy and asthmatic cats (Figure 2). One-way PERMANOVA confirmed a significant difference in the microbial community composition between the two groups (*p* = 0.0001; *F* = 10.03). Although the airways were predominated by the phyla *Proteobacteria, Bacteroidetes*, and *Firmicutes* in healthy and asthmatic cats, there were significant differences in the relative abundance of these taxa between groups. In healthy compared to asthmatic cats, changes were primarily attributed to a decreased relative abundance of *Pseudomonadaceae* (phylum *Proteobacteria*) from 78.3% ± 7.6% to 4.4% ± 0.3% (mean ± SEM); *p* < 0.001), and an increased relative abundance of *Moraxellaceae* (phylum *Proteobacteria*) from 3.0% ± 0.4% to 24.2% ± 0.1% (*p* < 0.001), *Weeksellaceae* (phylum *Bacteroidetes*) from 0.1% ± 0.01% to 52.4% ± 2.2 % (*p* < 0.001) and *Chitinophagaceae* (phylum *Bacteroidetes*) from 3.9% ± 1.0% to 22.5% ± 1.2 % (*p* < 0.001) (Figure 3). A volcano plot was used to identify the phyla and families that had a significant (*p* < 0.05) and at least a 2-fold change in relative abundance between health and asthma (Figure 4); 13 families met these criteria (Figure 5). The relative abundance of these taxa, as well as any taxa that were present in any cat at > 40% at any time point are summarized in Table 1.

**Table 1:** Relative abundance (mean ± SEM%) of taxa present in any BALF sample at a minimum of 0.5% in either health or asthma that underwent at least a 2-fold and significant change (*p* < 0.05) when comparing healthy and asthmatic cats or comprised more than 40% relative abundance in any sample.

**Phylum**								
**Family**								
**Genus Species**	**Health**			**Asthma**			***p*-value**	**Direction of change**
*Proteobacteria*	91.4	±	0.9	50.6	±	1.2	<0.001	▾
*Pseudomonadaceae*	78.3	±	0.8	2.0	±	0.1	<0.001	▾
*Pseudomonas*	73.5	±	0.8	0.2	±	0.02	<0.001	▾
*Sphingomonadaceae*	4.5	±	0.1	0.8	±	0.05	<0.001	▾
*Moraxellaceae*	3.0	±	0.1	24.7	±	1.0	0.004	▴
*Acinetobacter*	2.99	±	0.1	23.5	±	1.0	0.004	▴
*Xanthomonadaceae*	2.3	±	0.1	4.5	±	0.4	0.006	▴
*Stenotrophomonas*	0.0	±	0.0	2.5	±	0.5	0.003	▴
*Burkholderiaceae*	1.3	±	0.03	0.8	±	0.03	0.02	▾
*Xanthobacteraceae*	0.6	±	0.01	0.1	±	0.02	<0.001	▴
*Rhizobiaceae*	0.1	±	0.01	5.4	±	0.4	0.007	▴
*Nitrosomonadaceae*	0.0	±	0.0	1.0	±	0.1	0.01	▴
*Pasteurellaceae*	0.1	±	0.01	3.4	±	0.3	0.02	▴
*Bacteroidetes*	4.3	±	1.0	32.5	±	1.1	<0.001	▴
*Weeksellaceae*	0.1	±	0.01	0.8	±	0.05	0.039	▴
*Chitinophagaceae*	3.9	±	1.0	23.8	±	1.3	0.03	▴
*Filobacterium*	3.9	±	1.0	23.8	±	1.3	0.03	▴
*Muribaculaceae*	0.04	±	0.01	5.7	±	0.4	0.001	▴
*Porphyromonadaceae*	0.03	±	0.01	1.5	±	0.1	0.018	▴
*Flavobacteriaceae*	0.05	±	0.01	0.6	±	0.0	0.023	▴
*Sphingobacteriaceae*	0.1	±	0.004	0.05	±	0.0	0.017	▾
*Firmicutes*	1.8	±	0.1	7.9	±	0.4	0.0039	▴
*Streptococcaceae*	0.4	±	0.02	2.0	±	0.2	0.016	▴
*Staphylococcaceae*	0.4	±	0.02	1.1	±	0.01	0.004	▴
*Lachnospiraceae*	0.1	±	0.01	1.9	±	0.1	0.0009	▴
*Clostridiaceae 1*	0.1	±	0.01	0.2	±	0.02	0.033	▴
*Actinobacteria*	2.3	±	0.1	5.1	±	0.3	0.445	
*Propionibacteriaceae*	1.6	±	0.1	2.4	±	0.2	0.003	▴
*Corynebacteriaceae*	0.2	±	0.01	0.8	±	0.06	0.015	▴
*Microbacteriaceae*	0.2	±	0.03	1.8	±	0.2	0.022	▴
*Actinomycetaceae*	0.12	±	0.01	0.06	±	0.01	0.009	▾
*Fusobacteria*	0.15	±	0.01	1.02	±	0.1	0.0039	▴
*Fusobacteriaceae*	0.14	±	0.01	0.07	±	0.1	0.044	▾
*Tenericutes*	0.0	±	0.0	1.8	±	0.17	0.003	▴
*Mycoplasmataceae*	0.0	±	0.0	1.8	±	0.2	0.004	▴
*Mycoplasma felis*	0.0	±	0.0	1.7	±	0.2	0.004	▴
*Ureaplasma felinum*	0.0	±	0.0	0.1	±	0.01	0.092	

The arrows (▴, ▾) indicate the direction of the change in asthma relative to health.

### Intra-Group variation in community composition within asthmatic cats was observed

In comparison to healthy cats, in which *Pseudomonadaceae* (phylum *Proteobacteria*) was the dominating taxon, asthmatic cats had a more variable composition of their microbial communities. Sixty-one percent of asthmatic cats (16/26) had one predominant taxon, comprising more than 40% of the population. Relative abundance of *Filobacterium* spp. (family *Chitinophagaceae*) ranged from 48.1% to 97.1% in eight cats, *Acinetobacter* spp. (family *Moraxellaceae*) ranged from 42.4% to 90.4% in six cats, and there was one cat each with relative abundances of 59.2% *Stenotrophomonas* spp. (family *Xanthomonadaceae*) and *Pasteurellaceae* at 39.5%, respectively. The latter two cats had positive BALF cultures in agreement with the predominant taxa at the taxonomic level of ASV and family respectively (Supplementary Table 2). Additionally, four other cats had a predominance of *Acinetobacter* spp. ranging from 25.2% and 37.3% relative abundance.

### Potential pathogens were detected in asthmatic cats

*Filobacterium* spp. were sequenced in 2/11 (18%) healthy cats with relative abundances of 6.4 and 36.0% compared to 12/26 (46%) asthmatic cats, with a relative abundances of 2.2 to 97.1%. Family *Mycoplasmataceae*, was not sequenced in any healthy cat but was sequenced in 11/26 (42%) of asthmatic cats with relative abundance ranging from 0.1 to 24.2%, including the three cats with positive BALF cultures. Annotated to the ASV level, 5/11 cats had *Mycoplasma felis*, 2/11 had *Ureaplasma felinum*, and 4/11 had both taxa identified.

## Discussion

Spontaneous feline asthma, estimated to affect 1–5% of the pet cat population (36), is driven by T helper 2 cells, resulting in airway eosinophilia, airway hyperresponsiveness, and airway remodeling. It most closely resembles childhood-onset (type 2 high) asthma in humans (2) and has been proposed as a large animal model with relevance to One Health (22). The microbial composition and community structure of the lower airways of humans significantly differ between asthmatic and healthy states (17, 37) supporting the concept that respiratory dysbiosis occurs in human asthma. Similarly, in this study, respiratory dysbiosis was noted in spontaneously asthmatic cats and was characterized by decreased richness, increased α-diversity, and changes in the microbial community composition, including intra-group heterogeneity of predominating taxa and presence of taxa likely to be pathobionts or opportunistic pathogens. The respiratory microbiota may be influenced by environmental contributors (38), medications [antibiotics (39) or corticosteroids (40)], or even perturbations at distant mucosal sites such as the gut (41, 42). It is still unclear if altered microbial community composition is caused by or contributes to airway inflammation, but knowledge of altered bacterial populations in asthmatic airways could open the door for novel targeted therapies. Comprehensive characterization of microbial communities in the respiratory tract is only possible with sequencing, as evidenced by the high proportion of negative cultures in asthmatic cats corresponding to rich sequenced taxa. Compared to healthy cats and in parallel with human asthmatics (9, 17, 43), richness in asthmatic cats decreased. Many studies of human asthmatics cite inconsistent changes [increases (44, 45) or decreases (46)] in airway bacterial diversity as a key feature of dysbiosis compared with health. Diversity metrics vary across studies and include numbers of unique taxa present in each sample reflecting richness and evenness (α-diversity; Shannon and Simpson indices) or how many different taxa are shared between samples (β-diversity; Jaccard and Bray Curtis dis(similarity) indices). In spontaneously asthmatic cats compared to healthy cats, the Shannon and Simpson indices showed significantly increased alpha diversity. Taking into consideration the influence that different bacteria taxa may have on the microenvironment (e.g., microbial factors leading to disruption of immune maturation), β-diversity might provide a better insight into understanding the lower airway microbiota when comparing healthy and diseased states, especially when considering what taxa are significantly altered in disease or present in health and absent in disease. These changes in diversity may provide insight into which organisms are supportive of a “healthy” environment, potentially providing an avenue for intervention, in the way of replenishing or supporting the taxa associated with health. Using the Jaccard dissimilarity index, healthy and asthmatic cats had no overlap in microbial communities, and asthmatic cats had near obliteration of the *Pseudomonadaceae* family (phylum *Proteobacteria*), the predominant taxa found in healthy feline airways (27, 47). It is unclear if alterations in microbial community composition set the stage to initiate allergic airway inflammation culminating in spontaneous disease or if allergic asthma results in respiratory dysbiosis, further exacerbating and perpetuating the aberrant immune response. Certain identified pathobionts gaining a foothold in asthmatic airways, including *Filobacterium* and *Acinetobacter* spp., were noted in the current study. Additionally, *Mycoplasma felis*, a known lower airway pathogen of cats (48, 49) was identified in 35% of asthmatic but not in healthy cats, supporting that in addition to pathobionts, some opportunistic bacteria may also contribute to the respiratory dysbiosis observed in this study.

Asthma is a multifactorial heterogenous inflammatory airway disease classified both according to phenotype (clinical presentation) and endotype (distinct mechanistic pathways) (2, 50). While not universally recognized, two phenotypes are noted in cats, namely, cough variant only or cough in combination with clinical signs of airflow obstruction, including wheeze or episodic respiratory distress (51). Spontaneous feline asthma is orchestrated by T-helper 2 cells with allergen-specific IgE, leading to airway hyperresponsiveness and structural changes of the airways (22), modeling the atopic phenotype and T2 high endotype described in humans (2). Endotype characterization is important for therapy and prognosis and necessitates an understanding of a number of determinants, including the microbiome (50, 52, 53). Initial studies of the respiratory microbiota in humans showed the microbial composition and community structure of the lower airways were significantly different between asthmatic and healthy humans; however, these studies considered asthma as a single entity (17, 37, 54). More recently, the microbiota composition has been associated with disease severity and specific inflammatory pathways in humans with asthma underscoring the importance of recognizing phenotypic and endotypic characteristics (7, 55–57). Whether cats with different phenotypes have yet unrecognized differences in endotypes deserves further study with a more thorough immunologic evaluation but could be supported by the heterogeneity between bacterial taxa within the asthmatic cats of this study. The respiratory microbiota has been shown to help maintain mucosal homeostasis (10, 11) with microbiota being dynamic and competitive as they interact with the mucosal immune system (16). In health, the respiratory microbiota may directly inhibit pathogens from causing disease by a number of mechanisms, including competition for nutrients, biofilm formation, disruption of signaling molecules, direct bactericidal activity, and spatial occlusion (58). When homeostasis is disrupted by an infection, steroids, antibiotics, or other environmental triggers, a dysbiotic state can be established, which can foster a permissive environment for certain organisms to thrive and contribute to that dysbiotic state (10, 16). Certain commensal organisms exerting specific effects on the host mucosal immune system associated with the development of clinical disease are termed pathobionts. It has been postulated that treating dysbiosis by shifting the microbial community composition toward “health” or a homeostatic state may provide a more holistic approach than using antimicrobials to target an organism identified *via* standard culture techniques (16). While dysbiosis was demonstrated in all the asthmatic cats in this cohort, only 3 had positive bacterial cultures, highlighting the poor utility of this test alone to understand the impact of microbes in this lower airway disease of cats.

The majority of cats (69%) enrolled in this study had a predominance by sequencing of either *Filobacterium* spp. or *Acinetobacter* spp. *Filobacterium* spp., sometimes referred to as cilia-associated respiratory bacillus (CARB), has been recognized as a lower airway pathogen in several animal species (59–64). The presence of organisms consistent with CARB identified by light and electron microscopy was noted in a cat with an anesthetic death having bronchitis and bronchiolitis as well as in healthy cats (65), and in a larger group of cats with chronic bronchitis^35^. Identifying CARB as part of the healthy cat microbiota in a low relative abundance (3.9% ± 1.0%) and documenting the significant increase in relative abundance in asthmatic cats (23.8% ± 1.3%, *p* = 0.03), supports the concept of *Filobacterium* spp. as a pathobiont. Similarly, *Acinetobacter* spp., which has been detected in healthy research cats (66) and cats with experimentally induced asthma (1), were noted to have a significant increase in relative abundance when comparing healthy to asthmatic cats in the current study (2.99% ± 0.1% and 23.5% ± 1.0%, respectively; *p* = 0.004). Like *Filobacterium* spp., *Acinetobacter* spp. may be a pathobiont of the feline lower airways and perhaps changes in the lower airway microenvironment secondary to inflammation set the stage for them to thrive and potentially contribute to the pathogenesis of asthma. Mycoplasmas are considered commensals of the upper airways of cats and play a role in secondary infections of the upper respiratory tract and conjunctiva (67, 68). However, *Mycoplasma* spp. has not been detected in the lower airways of healthy cats using standard culture (69), PCR (23, 66), or microbiota analysis (1, 27, 47). In humans, infections with Mycoplasma can precede the onset of asthma, exacerbate symptoms and complicate the management of the disease, (70, 71) and successful treatment has led to improvement of lung function in asthmatic patients (72). While several studies have estimated the prevalence of Mycoplasma to be between 15%(48) and 35% (49) in cats with respiratory disease*, Mycoplasma* spp. has not been detected using sequencing in a cohort of research cats before or after experimental asthma induction (1). In contrast, it was detected in 9/26 (35%) of spontaneously asthmatic cats in the current study; 6 of which had moderate to severe clinical signs. While not technically fitting the definition of a pathobiont, as Mycoplasma is not a commensal symbiont of the lower airways, it stands to reason that either Mycoplasma is a true opportunistic infection, or it may populate the lower airways *via* extension from the upper airways to take advantage of an altered commensal environment (73). Considering the associations between *Mycoplasma* spp. detection and asthma in humans and the high prevalence of this study, testing for Mycoplasma in asthmatic cats is recommended as targeted therapy may improve clinical outcomes.

This study demonstrates dysbiosis of the lower respiratory microbiota in pet cats with spontaneous asthma and represents the first step in exploring the role that some taxa may play in the pathophysiology of feline asthma. Understanding of the precise effects of the lower airway microbiota on pulmonary health and the mechanisms by which it can influence or regulate the immune system is limited. Future studies including larger cohorts of pet cats with spontaneous asthma, by characterizing the microbiota with complimentary metabolomics and immune assays could further our knowledge in understanding the mechanisms by which the microbiota interacts with the host immune system. A larger cohort could allow for further characterization of the potential influence of steroids, antibiotics, or other comorbid conditions may have on the respiratory microbiota of asthmatic pet cats. This in turn may allow the development of novel therapeutic or management strategies in asthmatic cats which may serve as a large animal model for type 2 high asthma in humans.

## Data availability statement

The datasets presented in this study can be found in online repositories. The names of the repository/repositories and accession number(s) can be found in the article/Supplementary material.

## Ethics statement

The animal study was reviewed and approved by University of Missouri Animal Care and Use Committee.

## Author contributions

AV-P participated in the conception and design of the study, sample collection, DNA extraction, data analysis, interpretation, and drafted the manuscript. AE participated in the conception and design of the study, interpreted sequence data, and helped to draft the manuscript. ZM participated in the data analysis and contributed to the drafting of the manuscript. CR participated in the development of the animal model, the conception and design of the study, sample collection, data analysis, and helped to draft the manuscript. HR assisted with DNA extraction, sample collection, and study coordination. All authors contributed to the article and approved the submitted version.

## Conflict of interest

The authors declare that the research was conducted in the absence of any commercial or financial relationships that could be construed as a potential conflict of interest.

## Publisher's note

All claims expressed in this article are solely those of the authors and do not necessarily represent those of their affiliated organizations, or those of the publisher, the editors and the reviewers. Any product that may be evaluated in this article, or claim that may be made by its manufacturer, is not guaranteed or endorsed by the publisher.
