# Supplementary material for: Respiratory dysbiosis in cats with spontaneous allergic asthma
Source: Front Vet Sci. 2022 Sep 8;9:930385. doi: 10.3389/fvets.2022.930385 (PMC9492960; doi:10.3389/fvets.2022.930385)
Supplement: Supplementary file 1 [file Data_Sheet_1.PDF]

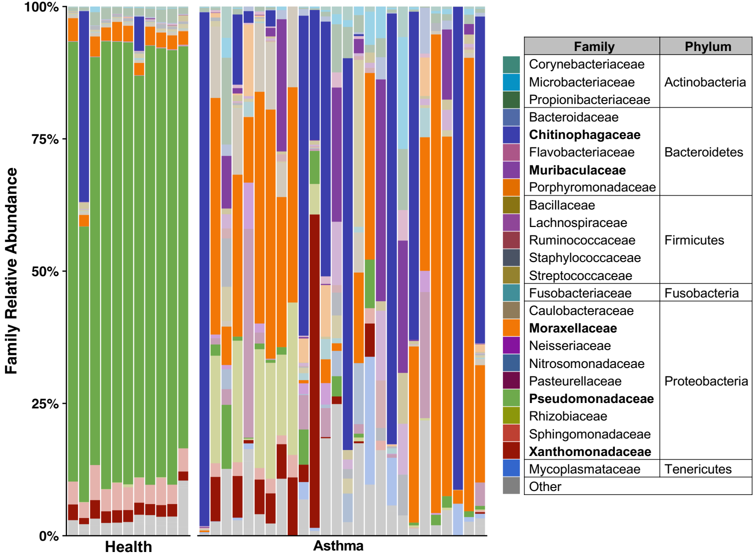
Family Relative Abundance
100%
75%
50%
25%
0%
Health
Asthma
Family
Phylum
Corynebacteriaceae
Microbacteriaceae
Propionibacteriaceae
Actinobacteria
Bacteroidaceae
Chitinophagaceae
Flavobacteriaceae
Muribaculaceae
Porphyromonadaceae
Bacteroidetes
Bacillaceae
Lachnospiraceae
Ruminococcaceae
Staphylococcaceae
Streptococcaceae
Firmicutes
Fusobacteriaceae
Fusobacteria
Caulobacteraceae
Moraxellaceae
Neisseriaceae
Nitrosomonadaceae
Pasteurellaceae
Pseudomonadaceae
Rhizobiaceae
Sphingomonadaceae
Xanthomonadaceae
Proteobacteria
Mycoplasmataceae
Tenericutes
Other
