# Supplementary figures and images for: Respiratory dysbiosis in cats with spontaneous allergic asthma
Source: Front Vet Sci. 2022 Sep 8;9:930385. doi: 10.3389/fvets.2022.930385 (PMC9492960; doi:10.3389/fvets.2022.930385)

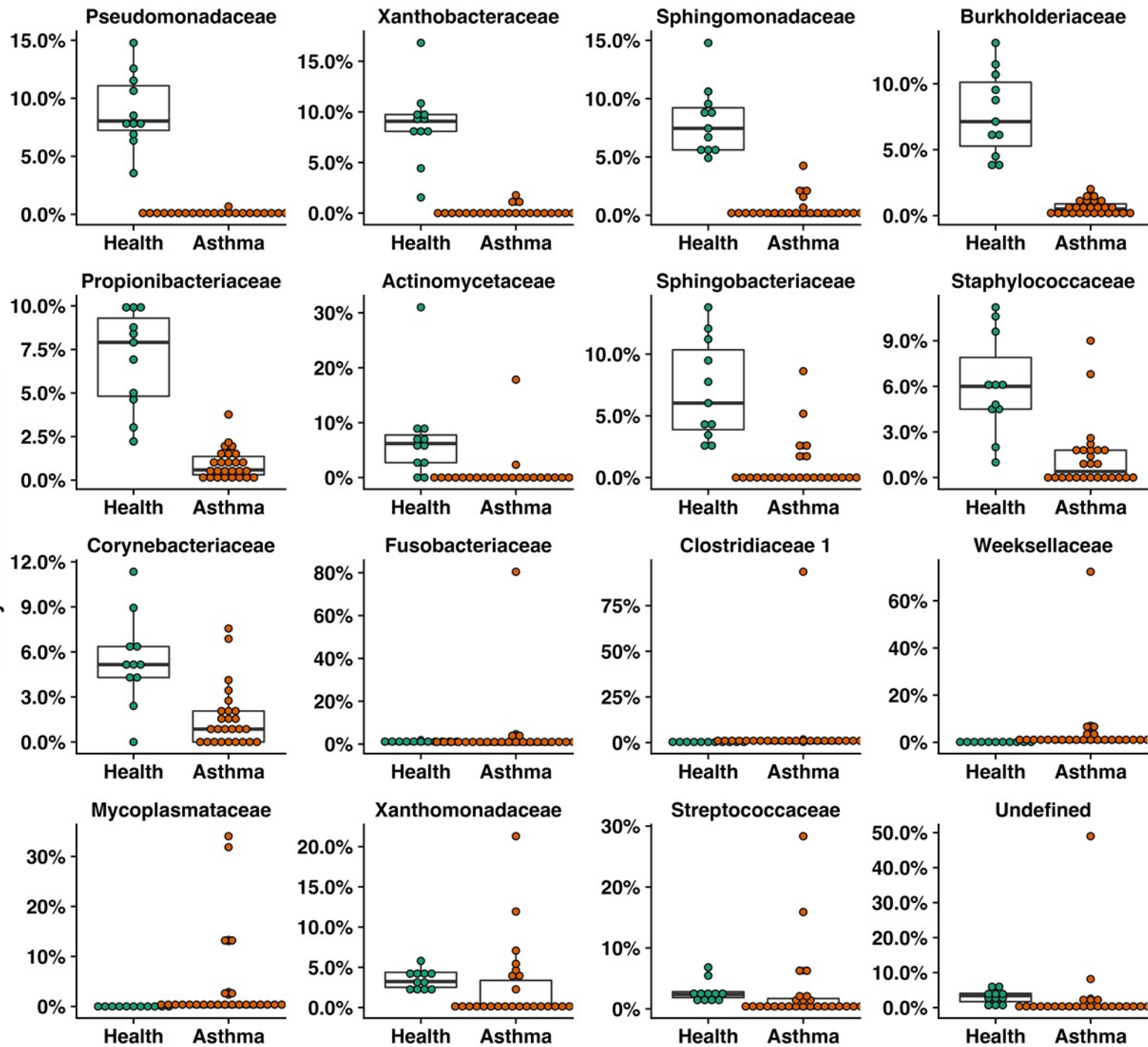

Supplement: Supplementary file 2 [file Data_Sheet_2.PDF]
