# Supplementary material for: Respiratory dysbiosis in cats with spontaneous allergic asthma
Source: Front Vet Sci. 2022 Sep 8;9:930385. doi: 10.3389/fvets.2022.930385 (PMC9492960; doi:10.3389/fvets.2022.930385)
Supplement: Supplementary file 3 [file Data_Sheet_3.docx]

| Cat | Age | Gender | BCS | Environment | CSS | %BALF  eos | %BALF  pmns | Antibiotic | Steroid | Predominant  sequenced taxa | (%RA) | *Myco* (%RA) |
| --- | --- | --- | --- | --- | --- | --- | --- | --- | --- | --- | --- | --- |
| 1 | 12 | FS | 5 | Indoor | 1 | 14 | 10 | N | N | *Filobacterium* spp. | 61.76 | 0.87 |
| 2 | 6 | FS | 9 | Both | 3 | 23 | 14 | N | Y | *Stenotrophomonas* | 59.22 | 0.59 |
| 3 | 3 | FS | 7 | Both | 1 | 67 | 1 | Y | N | *Streptococcus* spp. | 31.53 | 0 |
| 4 | 5 | FS | 6 | Both | 2 | 22 | 25 | N | N | *Filobacterium* spp. | 60.47 | 3.29 |
| 5 | 4 | MC | 5 | Both | 2 | 47 | 22 | N | N | *Acinetobacter* spp. | 85.68 | 0.88 |
| 6 | 2 | FS | 4 | Both | 2 | 55 | 3 | Y | Y | *Filobacterium* spp. | 74.04 | 0 |
| 7 | 7 | MC | 7 | Indoor | 1 | 18 | 2 | N | N | *Acinetobacter* spp. | 46.23 | 0 |
| 8 | 6 | MC | 5 | Both | 1 | 45 | 17 | Y | N | *Acinetobacter* spp. | 25.17 | 0 |
| 9 | 4 | MC | 6 | Both | 3 | 15 | 49 | N | N | *Acinetobacter* spp. | 35.27 | 24.15 |
| 10 | 2 | FS | 6 | Indoor | 1 | 64 | 11 | N | N | *Acinetobacter* spp. | 37.29 | 0 |
| 11 | 7 | MC | 6 | Indoor | 3 | 15 | 2 | N | Y | *Family Pasturellaceae* | 13.43 | 0 |
| 12 | 5 | MC | 5 | Indoor | 1 | 40 | 44 | N | N | *Microbacterium sp.* | 15.38 | 0 |
| 13 | 8 | FS | 4 | Indoor | 1 | 23 | 1 | Y | N | *Acinetobacter* spp. | 43.36 | 0.11 |
| 14 | 5 | MC | 3 | Indoor | 1 | 56 | 4 | N | N | *Family Muribaculaceae* | 11.48 | 0 |
| 15 | 1.5 | MC | 7 | Both | 3 | 54 | 36 | N | N | *Filobacterium* spp. | 91.28 | 5.94 |
| 16 | 10 | MC | 7 | Indoor | 1 | 11 | 17 | N | N | *Filobacterium* spp. | 97.2 | 0.2 |
| 17 | 2 | MC | 5 | Indoor | 3 | 28 | 2 | N | N | *Acinetobacter* spp. | 29.79 | 0.49 |
| 18 | 10 | FS | 5 | Both | 3 | 26 | 61 | N | N | *Acinetobacter* spp. | 42.42 | 0 |
| 19 | 6 | FS | 5 | Indoor | 2 | 65 | 15 | N | N | *Acinetobacter* spp. | 90.44 | 0 |
| 20 | 9 | MC | 4 | Indoor | 1 | 45 | 44 | N | N | *Filobacterium* spp. | 81.39 | 9 |
| 21 | 8 | MC | 7 | Indoor | 2 | 25.5 | 37 | Y | Y | *Cutibacterium* spp. | 16.4 | 0 |
| 22 | 1.5 | MC | 4 | Both | 3 | 71 | 9 | N | N | *Family Pasteurellaceae* | 39.46 | 1.08 |
| 23 | 1 | FS | 7 | Both | 2 | 22 | 14 | N | Y | *Filobacterium* spp. | 62 | 0 |
| 24 | 9 | FS | 4 | Indoor | 1 | 65 | 7.5 | N | N | *Family Muribaculaceae* | 11.48 | 0 |
| 25 | 3 | FS | 4 | Both | 1 | 32 | 11 | Y | N | *Filobacterium* spp. | 48.14 | 0 |
| 26 | 1.5 | MC | 7 | Indoor | 2 | 36 | 12 | N | Y | *Acinetobacter* spp. | 67.95 | 0 |

Supplementary table 1. Individual cat data including clinical data and description of most abundant taxa as well as presence or absence of *Mycoplasmacetae*. FS – female spayed, MC – male castrated, BCS – body condition score, CSS – clinical severity score (1 = mild (cough alone); 2 = moderate (wheezing or exercise intolerance); or 3 = severe (respiratory distress episodes), BALF eos – percent eosinophils in bronchoalveolar lavage fluid, BALF pmns – percent polymorphonuclear cells or neutrophils in bronchoalveolar lavage fluid, Y – yes, N – no, %RA – percent relative abundance, Myco – Family *Mycoplasmacetaceae*

| Cat # | Organism in Culture | Closest 16S RNA match | % RA | Predominant taxa | % RA |
| --- | --- | --- | --- | --- | --- |
| 2 | *Stenotrophomonas maltophilia* | *Stenotrophomonas* spp. | 59.2 | *Stenotrophomonas* spp. | 59.2 |
|  | *Pseudomonas putida* | *Pseudomonas* spp. | 6.2 |  |  |
| 4 | *Actinobacillus*/*Pasteurella* group | *Pasteurellaceae* | 6.5 | *Chitinophagaceae* | 60.5 |
| 22 | *Pasteurella multocida* | *Pasteurellaceae* | 39.5 | *Pasteurellaceae* | 39.5 |
|  | *Streptococcus* spp. | *Streptococcus* spp. | 0.6 |  |  |
|  | *Pseudomonas aeruginosa* | *Pseudomonas* spp. | 0.17 |  |  |

Supplementary table 2. Bronchoalveolar lavage fluid bacterial culture results correlated to sequencing data. %RA – percent relative abundance
